# Interchangeability of the Assays Used to Assess the Activity of Anti-SARS-CoV-2 Monoclonal Antibodies

**DOI:** 10.3390/v15081698

**Published:** 2023-08-05

**Authors:** Brady T. Hickerson, Alexey M. Khalenkov, Tao Xie, David M. Frucht, Dorothy E. Scott, Natalia A. Ilyushina

**Affiliations:** 1 Division of Biotechnology Review and Research II, Center for Drug Evaluation and Research, Food and Drug Administration, Silver Spring, MD 20993, USA; brady.hickerson@fda.hhs.gov (B.T.H.); tao.xie@fda.hhs.gov (T.X.); david.frucht@fda.hhs.gov (D.M.F.); 2 Division of Plasma Derivatives, Center for Biologics Evaluation and Research, Food and Drug Administration, Silver Spring, MD 20993, USA; alexey.khalenkov@fda.hhs.gov (A.M.K.); dorothy.scott@fda.hhs.gov (D.E.S.)

**Keywords:** monoclonal antibody, antiviral activity, potency, neutralization assay, ELISA, surface plasmon resonance, Bland–Altman analysis, method interchangeability

## Abstract

The recent global COVID-19 pandemic caused by SARS-CoV-2 lasted for over three years. A key measure in combatting this pandemic involved the measurement of the monoclonal antibody (mAb)-mediated inhibition of binding between the spike receptor-binding domain (RBD) and hACE2 receptor. Potency assessments of therapeutic anti-SARS-CoV-2 mAbs typically include binding or cell-based neutralization assays. We assessed the inhibitory activity of five anti-SARS-CoV-2 mAbs using ELISA, surface plasmon resonance (SPR), and four cell-based neutralization assays using different pseudovirus particles and 293T or A549 cells expressing hACE2 with or without TMPRSS2. We assessed the interchangeability between cell-based and binding assays by applying the Bland–Altman method under certain assumptions. Our data demonstrated that the IC_50_ [nM] values determined by eight neutralization assays are independent of the cell line, presence of TMPRSS2 enzyme on the cell surface, and pseudovirus backbone used. Moreover, the Bland–Altman analysis showed that the IC_50_ [nM] and K_D_ [nM] values determined by neutralization/ELISA or by SPR are equivalent and that the anti-spike mAb activity can be attributed to one variable directly related to its tertiary conformational structure conformation, rate dissociation constant K_off_. This parameter is independent from the concentrations of the components of the mAb:RBD:hACE2 complexes and can be used for a comparison between the activities of the different mAbs.

## 1. Introduction

The COVID-19 pandemic caused by the severe acute respiratory syndrome coronavirus 2 (SARS-CoV-2) lasted for more than three years and has infected over 750 million people worldwide [1,2]. SARS-CoV-2 entry into cells starts with binding of the viral spike glycoprotein to the human angiotensin-converting enzyme 2 (hACE2) receptor. There are two mechanisms of viral entry into the cytosol: (1) viral particles are internalized by endocytosis and release viral RNA into the cytosol by fusion of the viral envelope with the endosome membrane after exposure to the acidic environment of the endosome; or (2) the viral spike protein binds to the hACE2 receptor and undergoes conformational changes driven by transmembrane protease serine 2 (TMPRSS2), followed by fusion of the viral envelope and cell membrane resulting in releasing the viral RNA into the cytosol [3].

A key measure in combatting the COVID-19 pandemic involved the development of antibody-based therapeutics geared toward inhibiting viral spike binding to hACE2 [4,5,6,7]. Several monoclonal antibodies (mAbs) and mAb combinations that target the SARS-CoV-2 spike protein received emergency use authorization (EUA) from the FDA for use as pre- or post-exposure prophylaxis and/or treatment of COVID-19. These included bebtelovimab, tixagevimab combined with cilgavimab, sotrovimab, bamlanivimab combined with etesevimab, and casirivimab combined with imdevimab [8]. Although highly effective against early SARS-CoV-2 variants, these products are not currently authorized in the United States due to the emergence and widespread circulation of variants that are resistant to neutralization by these mAbs in cell culture [9,10,11]. Therefore, the continued development of antivirals against SARS-CoV-2 infection is urgently needed.

Research involving SARS-CoV-2, which is a biosafety level (BSL)-3 virus requires BSL-3 facilities. The high cost and limited availability of BSL-3 facilities impedes the rapid and successful development of new therapeutic candidates. Therefore, BSL-2-based assays are frequently used to assess the potency of therapeutic anti-SARS-CoV-2 mAbs targeting the spike glycoprotein. These assays typically include direct or indirect binding assays (enzyme-linked immunosorbent assay (ELISA) or a surface plasmon resonance (SPR)) or a cell-based neutralization assay [12,13], which uses either viral spike glycoprotein (or its receptor-binding domain, RBD) or pseudotype viral particles bearing spike protein, respectively [12,13,14,15,16]. While ELISA and SPR assays are used to measure the concentrations and binding affinities of the mAbs to selected spike epitopes, the cell-based neutralization assay provides a more comprehensive assessment of the mAb-meditated antiviral activity by measuring the inhibition of SARS-CoV-2 cellular entry [12,13]. A wide variety of pseudotyped virus particles and cell lines have been used to assess the potency of anti-SARS-CoV-2 mAbs with antiviral activity. Commonly used reagents include retroviral (HIV-1 or murine leukemia virus (MLV)-derived) or rhabdoviral (VSV)-based packaging vector systems and immortalized human cells (embryonic kidney 293T or human lung carcinoma epithelial (A549) cells) expressing hACE2 with or without co-expression of TMPRSS2 [9,17,18].

Previous studies have assessed the degree of correlation between ELISA and cell-based neutralization assays. While the ELISA assay does not require the use of infectious virus particles, data obtained from the ELISA binding have not always correlated well with results obtained from cell-based neutralization assays or studies using authentic SARS-CoV-2 isolates [12,15,19]. In contrast, cell-based neutralization assays using lentivirus (LV) or MLV pseudovirus particles expressing the SARS-CoV-2 spike glycoprotein have produced results that correlated well with those obtained from neutralization assays using the authentic SARS-CoV-2 virus [12,14,20]. However, since correlation analysis usually estimates the linear relationship between two independently measured variables, but not the differences in two methods used to measure a single variable, it is poorly suited for the assessment of interchangeability between different methods employed for measurement of the mAb antiviral activity [21]. In this study, we assessed the inhibitory activity of the five anti-SARS-CoV-2 mAbs using two binding assays (ELISA and SPR) and four cell-based neutralization assays using LV or MLV pseudovirus particles (pLV and pMLV) expressing SARS-CoV-2 Wuhan, Delta, or Omicron spike glycoprotein and 293T or A549 cells expressing hACE2 with or without TMPRSS2. We provide a detailed analysis of results from activity measurements of the selected mAbs determined by each method. We also report the comparison between different methods as calculated by the Bland–Altman method comparability analysis under certain assumptions [22].

## 2. Materials and Methods

### 2.1. Cells, mAbs, and Plasmids

Human embryonic kidney (293T) cells were obtained from American Tissue Culture Collection (Manassas, VA, USA) and were maintained as previously described [23]. The 293T cells stably expressing hACE2 with or without TMPRSS2 (293T-hACE2 and 293T-hACE2-TMPRSS2) and A549 cells stably expressing hACE2 (A549-hACE2) were obtained through BEI Resources, National Institute of Allergy and Infectious Diseases, NIH: NR-52511, NR-55293, and NR-53821. The A549 cells stably expressing hACE2 and TMPRSS2 (A549-hACE2-TMRPSS2) were purchased from Invivogen (San Diego, CA, USA). All cell lines were cultured in Dulbecco’s modified eagle medium supplemented with 10% fetal bovine serum at 37 °C and 5% CO_2_.

Casirivimab, imdevimab, bamlanivimab, and etesevimab were obtained from Invivogen. SARS-CoV-2 (Omicron) neutralizing antibody standard (#A02161, referred to as GenScript standard throughout the manuscript) was purchased from GenScript (Piscataway, NJ, USA). Plasmid pCMV delta R8.2 was a gift from Didier Trono (Addgene plasmid #12263), plasmid gag/pol was a gift from Tannishtha Reya (Addgene plasmid #14887), and plasmids pBOBI-FLuc, pCMV-Fluc, pcDNA3.3_CoV2-D18, pcDNA3.3_SARS2_B.1.617.2, and pcDNA3.3_SARS2_omicron_BA.1 were a gift from David Nemazee (Addgene plasmids #170674, #170575, #170442, #172320, #180375).

### 2.2. Pseudovirus Production and Neutralization Assays

The pLV and pMLV bearing SARS-CoV-2 spike glycoprotein and carrying a firefly luciferase reporter gene were produced in 293T cells. Briefly, two plasmids (pCMV delta R8.2, pBOBI-FLuc) or two plasmids (gag/pol and pCMV-Fluc) and a plasmid expressing the SARS-CoV2 spike protein (pcDNA3.3_CoV2-D18, pcDNA3.3_SARS2_B.1.617.2, or pcDNA3.3_SARS2_omicron_BA.1 of the Wuhan, Delta, or Omicron strains, respectively) were co-transfected in 293T cells to generate pLV or pMLV particles, respectively. Pseudovirus supernatants were collected approximately 48 h post-transfection, filtered through a 0.45 μm low protein binding filter, and used immediately or stored at −80 °C. Pseudovirus titers were measured by infecting specific target cells (seeded at a cell density of ≈5 × 10^4^ cells/well) for 72 h prior to measuring luciferase activity (Promega, Madison, WI, USA), as described previously [16]. Pseudovirus titers were expressed as relative luminescence unit per milliliter of pseudovirus supernatants (RLU/mL).

Neutralization assays were performed in A549 or 293T cells transiently expressing hACE2 with or without co-expression of TMPRSS2. Briefly, pseudoviruses expressing the SARS-CoV-2 spike protein (or mixtures of pseudoviruses expressing different spike proteins) with titers of approximately 2 × 10^4^ RLU/mL of luciferase activity were incubated with mAbs for one hour at 37 °C. Pseudovirus and antibody mixtures (100 μL) were then inoculated onto 96-well plates that were seeded with 5 × 10^4^ cells/well one day prior to infection. Pseudovirus infectivity was scored 72 h later for luciferase activity. The mAb concentration causing a 50% reduction in RLU compared to control was defined as the inhibitory concentration 50% (IC_50_). Values were calculated using a nonlinear regression curve fit (GraphPad Prism software Inc., La Jolla, CA, USA). The mean mAb concentration resulted in a 50% reduction in the RLU compared to control from at least two independent experiments that was reported as the final IC_50_ value.

### 2.3. Droplet Digital PCR (ddPCR)

Droplet Digital PCR was used to determine the ratios of the LV pseudovirus particles (Wuhan:Delta, Wuhan:Omicron, and Delta:Omicron) used in the neutralization assay. Stocks of the pLV particles expressing the SARS-CoV-2 Wuhan, Delta, or Omicron spike variants were mixed at ratios of 100%:0%, 90%:10%, 75%:25%, 50%:50%, 25%:75%, 10%:90%, and 0%:100% to achieve approximately 20,000 RLU/100 µL. RNA was then extracted from the mixtures using the Qiagen RNeasy Mini kit (Germantown, MD, USA) and ddPCR was performed using the harvested RNA samples with the BioRad one-step ddPCR advanced kit for probes (Hercules, CA, USA) according to the manufacturer’s instructions. The primers and probes targeting the SARS-CoV-2 spike gene were designed using the DNASTAR Lasergene software (Integrated DNA Technologies, Coralville, IA, USA) and are available upon request. Droplets were generated using the BioRad automated droplet generator and the ddPCR run was performed using the BioRad C1000 thermal cycler using the following protocol: 60 °C for 30 min, 95 °C for 10 min, and then 40 cycles at 95 °C for 30 s and 56 °C for 1 min, followed by a post-cycle step at 98 °C for 10 min and an infinite hold at 4 °C. The droplets were then transferred to the BioRad QX200 reader, analyzed with the FAM (Wuhan and Delta) and VIC (Delta and Omicron) channels, and the data were visualized using the BioRad QuantaSoft program. No-template control reactions were used as references to set thresholds. The results were then used to adjust the input volume of the pLV stocks to achieve the desired ratios.

### 2.4. ELISA

The mAbs’ activity against the SARS-CoV-2 Wuhan, Delta, and Omicron spike proteins were determined using the SARS-CoV-2 surrogate virus neutralization test kit obtained from GenScript according to the manufacturer’s instructions. Briefly, mAbs were serially diluted 1:10 before being mixed with an equal volume of SARS-CoV-2 Wuhan, Delta, or Omicron spike protein conjugated to horseradish peroxidase. The mixtures were incubated for 30 min at 37 °C and then transferred to microtiter plate coated with hACE2 receptor. After incubation for 15 min at 37 °C, the plate was washed, and 100 µL of 3,3′,5,5′-tetramethylbenzidine (TMB) solution was added. The reaction was stopped with TMB stop solution after 15 min and the optical density was determined at 450 nm. The mAb concentration that caused a 50% reduction in 450 nm absorbance values compared to negative controls was defined as the IC_50_. Two independent experiments were performed at least in duplicate, and positive and negative controls and mAb GenScript standard were included in each test to determine the validity of the test.

### 2.5. SPR Assay

Biacore T200 biosensor (Cytiva, Sweden) was used to determine kinetic and steady-state dissociation (K_D_ [nM]) and affinity constants (K_A_ = 1/K_D_) for the interaction between mAbs and recombinant RBD epitopes. Briefly, mouse anti-human Fc mAb was covalently immobilized on the dextran layer of the CM5 chip (#BR100399) using human antibody capture kit reagents (#BR100839) according to the manufacturer’s instructions. To determine the K_D_, every mAb was diluted in HBS-EP + buffer (#BR100826) and then captured via Fc interaction with the anti-Fc immobilized mAb at the levels projecting the maximum analyte’s response of ≈100 RU. RBDs (#40150-V08B2, #40592-V08H90, and #40592-V08H121 for Wuhan, Delta, and Omicron, respectively (SinoBiological, Wayne, PA, USA)) were first serially diluted in HBS-EP+ buffer and then applied at the flow rate of 75 µL/min. The association and dissociation times were restricted to 120 s and to 1200 sec, respectively, and all experiments were carried out at 25 °C. Chip was regenerated each cycle by 30 s pulse with regeneration solution (3M MgCl). Sensograms were normalized for both blank and non-specific binding signals and analyzed using Biacore T200 Evaluation software v.2.1. by fitting to the 1:1 Langmuir interaction model. The kinetic dissociation constant K_D_ [nM] was calculated as a ratio of rate constants K_off_ [s^−1^]/K_on_ [nM^−1^s^−1^]. Subsequently, the association constant K_A_ can be defined as a reciprocal of equilibrium K_D_ or as a ratio of rate constants. The steady-state K_D_ was measured as a concentration of recombinant RBD at equilibrium R_eq_ = R_max_/2. The half-life of the mAb:RBD complex was calculated as T_1/2_ [s] = ln(2)/K_off_ [24].

### 2.6. Statistical Analysis

ELISA, SPR, and neutralization assays were compared by the Bland–Altman analysis, i.e., by plotting differences between measurements obtained by two methods vs. the mean of the two measurements obtained by these methods [22]. The bias between two assays was calculated as an average of the differences. Two assays were regarded as interchangeable if the bias between these methods was not statistically different from 0.

## 3. Results

**Figure 1:**
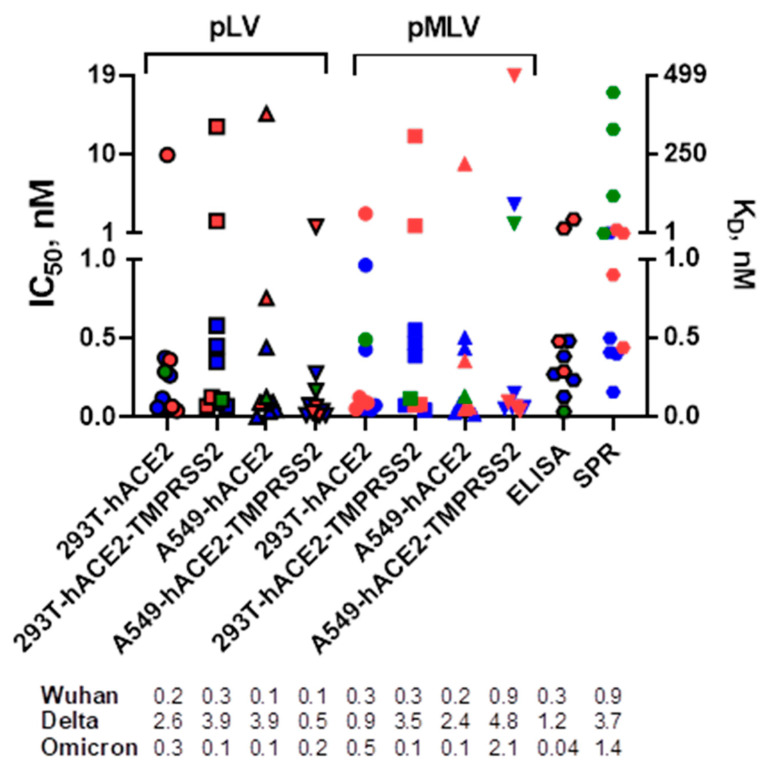
Neutralization of pLV and pMLV particles by mAbs as measured by neutralization assays in 293T and A549 cells expressing ACE2 with and without TMPRSS2 and binding of the mAbs to SARS-CoV-2 Wuhan (blue), Delta (red), and Omicron (green) variants by ELISA and SPR assays. Various symbols are used to distinguish different assays. For Wuhan and Delta variants, the mean IC_50_ [nM] and K_D_ [nM] values are calculated based on the data from all five mAbs for neutralization/ELISA and SPR assays, respectively. For Omicron variant, the IC_50_ [nM] and K_D_ [nM] values are indicated for the GenScript standard only.

We first compared the neutralization profiles of five mAbs, casirivimab, imdevimab, bamlanivimab, etesevimab, and GenScript standard, against pLV and pMLV particles expressing the SARS-CoV-2 Wuhan, Delta, or Omicron spike glycoprotein in 293T or A549 cells expressing hACE2 with or without TMPRSS2 (Figure 1). All mAbs displayed neutralization activities against SARS-CoV-2 Wuhan and Delta (IC_50_ values ranged between 0.003 and 18.9 nM), except for bamlanivimab that did not show measurable neutralization titers against the Delta variant. Only the GenScript standard potently neutralized all three SARS-CoV-2 spike variants. These results correlated well with the previously published studies by others [10,25,26]. We next determined IC_50_ values by the ELISA assay and observed that the mean titers were similar to those found in microneutralization assays. As seen by SPR assay (Figure 1), all mAbs were able to bind SARS-CoV-2 spikes (K_D_ values ranged between 0.2 and 446 nM), except for GenScript standard and imdevimab that did not bind Delta and Omicron variants, respectively.

Since four mAbs (casirivimab, imdevimab, bamlanivimab, and GenScript standard) showed the highest activity (i.e., lowest IC_50_ values) against the Wuhan variant (i.e., “primary” variant) and one mAb (etesevimab) showed the highest neutralization activity against the Delta variant across three SARS-CoV-2 variants, we next assessed the mAb neutralization titers against the pseudovirus mixtures containing pLV particles expressing different SARS-CoV-2 variants at different ratios (“primary”:“secondary”, Wuhan:Delta, Wuhan:Omicron, or Delta:Omicron at 100%:0%, 90%:10%, 75%:25%, 50%:50%, 25%:75%, 10%:90%, and 0%:100%) and determined the abundance of pLVs expressing the “secondary” SARS-CoV-2 spike variant present in the mixtures needed to decrease by 50% the mAb IC_50_ value against the “primary” variant. We observed that the majority of the mAbs can exert at least 50% of their activity against pLV mixtures containing Wuhan:Delta and Wuhan:Omicron variants if the mixtures contained ≥46% of the pseudovirus particles expressing the SARS-CoV-2 Wuhan “primary” variant (Table 1).

**Figure 2:**
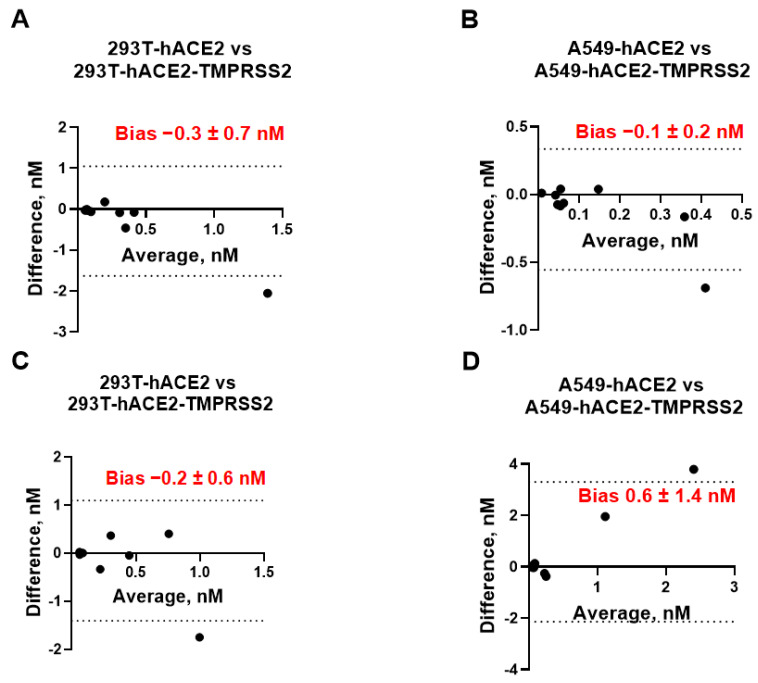
Bland–Altman plots showing equivalency of neutralization assays performed in 293T-hACE2 vs. 293T-hACE2-TMPRSS2 cells (**A**,**C**) or A549-hACE2 vs. A549-hACE2-TMPRSS2 cells (**B**,**D**) using pLV (**A**,**B**) or pMLV (**C**,**D**) particles. Bias [nM] between each pair of neutralization assays is indicated. Dashed lines represent 95% confidence interval (CI) for bias.

We next assessed whether cells with or without TMPRSS2 can be used interchangeably for measuring the mAb neutralization titers. To investigate any possible relationship between the measurement error and the true value, we plotted the difference between the IC_50_ values measured in cells expressing hACE2 vs. the same cell line expressing both receptors (293T/A549-hACE2 vs. 293T/A549-hACE2-TMPRSS2) against their mean (Figure 2). We did not observe any considerable discrepancies between each pair of neutralization assays. The bias (average of the differences) between assays ranged between −0.3 nM to +1.6 nM at a 95% confidence interval (CI) and was not significantly different from 0, indicating that the IC_50_ values measured by neutralization assays in cell lines with and without TMPRSS2 are essentially equivalent.

**Figure 3:**
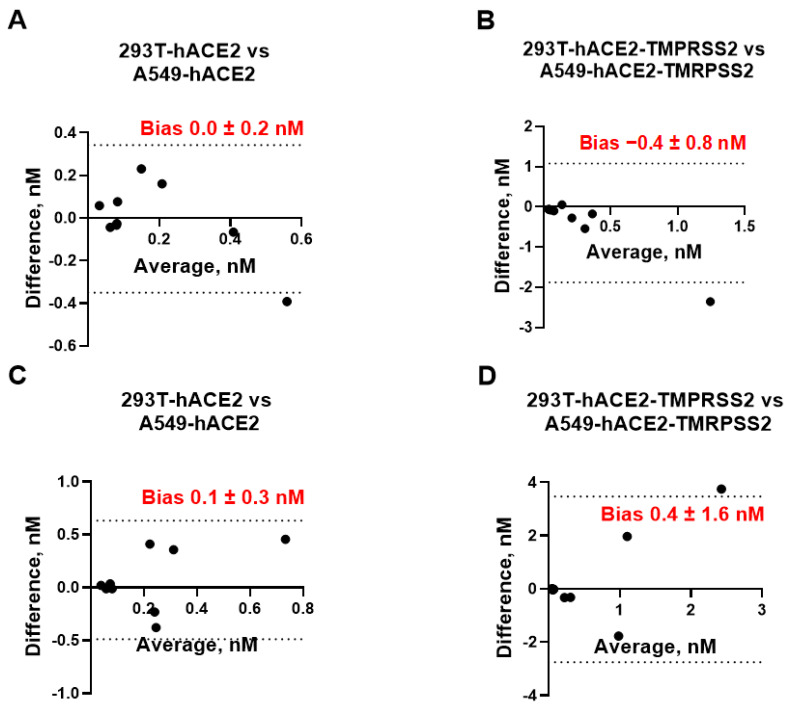
Bland–Altman plots showing equivalency of neutralization assays performed in 293T-hACE2 vs. A549-hACE2 cells (**A**,**C**) or 293T-hACE2-TMPRSS2 vs. A549-hACE2-TMPRSS2 cells (**B**,**D**) using pLV (**A**,**B**) or pMLV (**C**,**D**) particles. Bias [nM] between each pair of neutralization assays is indicated. Dashed lines represent 95% confidence interval (CI) for bias.

**Figure 4:**
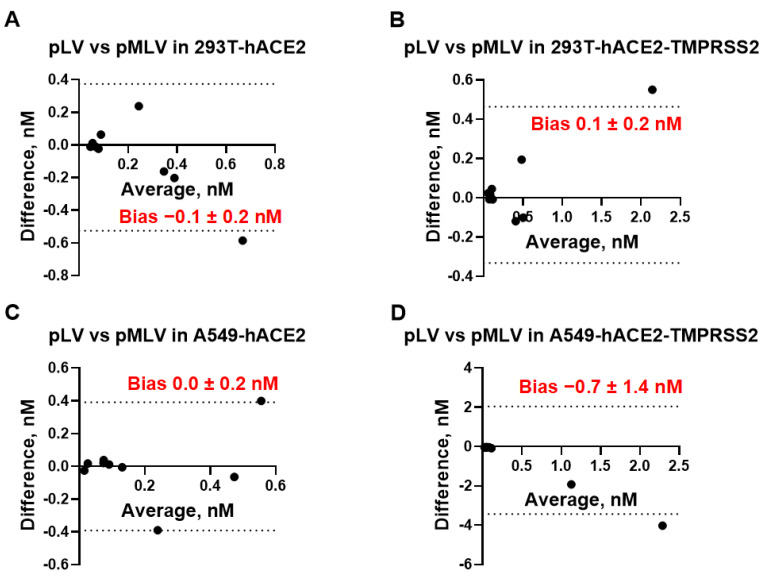
Bland–Altman plots showing equivalency of neutralization assays using pLV vs. pMLV particles and performed in 293T-hACE2 (**A**), 293T-hACE2-TMPRSS2 (**B**), A549-hACE2 (**C**), and A549-hACE2-TMPRSS2 cells (**D**). Bias [nM] between each pair of neutralization assays is indicated. Dashed lines represent 95% confidence interval (CI) for bias.

We also assessed whether 293T and A549 cells can be used interchangeably for measuring mAb neutralization titers. After plotting the difference between the IC_50_ values measured in 293T-hACE2 ± TMPRSS2 cells vs. A549-hACE2 ± TMPRSS2 against the respective mean value (Figure 3), we found that the bias between each pair of the assays was not significantly different from 0 and ranged between −0.4 nM and +0.4 nM at a 95% CI, suggesting that neutralization assays employing 293T cells expressing hACE2 with or without TMPRSS2 can be replaced by A549 cells expressing the same receptors without affecting IC_50_ measurements. We also assessed whether pLV and pMLV particles can be used interchangeably for measuring mAb neutralization titers. As seen in Figure 4, bias values did not differ significantly from 0, indicating that the IC_50_ values measured by using pLV or pMLV particles are essentially equivalent in each cell line studied and both backbones are interchangeable. Taken together, IC_50_ [nM] values determined by eight different neutralization assays were found to be equivalent and every assay can be replaced by any other neutralization assay without affecting accurate measurement of the mAb activity.

Finally, we applied the Bland–Altman analysis for the comparison of the cell-based and binding (ELISA and SPR) assays (Table 2). We used IC_50_ [nM] or K_D_ [nM] values as a measurement of the mAb activity by neutralization/ELISA or the SPR method, respectively. Notably, bias values between each pair of the assays did not differ significantly from 0 at a 95% CI, indicating the equivalency of all the methods between each other and equivalency of IC_50_ [nM] to K_D_ [nM] values.

## 4. Discussion

We studied the interaction of five commercially available anti-SARS-CoV-2 mAbs with the viral spike proteins (RBDs) by three different methods, cell-based pseudovirus neutralization assay, competition ELISA, and SPR assay. To assess the interchangeability of these methods, we used three SARS-CoV-2 spike proteins, two pseudovirus backbones, and two cell lines expressing hACE2 with and without the TMPRSS2 co-receptor. While we did not estimate the concentration or distribution of SARS-CoV-2 spike proteins or the hACE2 receptors on the surface of the pseudovirus particles or cells, respectively, nor the ratio of the pseudovirus:cells needed for the pseudovirus entry to occur, we implied that during the 72-h incubation time each uninhibited pseudovirus particle would get endocytosed. We also assumed that each pseudovirus particle contains more than one spike protein on its surface and that an interaction of 1:1 RBD:hACE2 results in pseudovirus entry into the cell leading to infection. An additional assumption was that one mAb molecule can block one RBD and that the formation of a 1:1 mAb:RBD complex prevents RBD from binding to hACE2 while this complex exists. Under all these assumptions, we demonstrated with the Bland–Altman analysis that the IC_50_ [nM] values determined by eight different neutralization assays are independent of the cell line, presence of the TMPRSS2 receptor on the cell surface, and pseudovirus backbone used. This finding is in a good agreement with the previous study by Riepler et al. [27], who found that results from four different neutralization assays correlated well with each other.

Since similar IC_50_ values measured by neutralization assays and competition ELISA were observed and since these methods were found to be essentially equivalent for measuring mAb IC_50_ values, factors such as pseudovirus backbones, the RBD/spike surface distribution, ratio of infectious to inhibited pseudovirus particles, and type of the cell lines (except hACE2 expression at appropriate concentrations) can be excluded from factors that may have impacted the mAb IC_50_ [nM] values in our study. We can speculate that inhibitory mAb:RBD interaction occurs in the extracellular compartment before the virus can be internalized independent of the virus cell entry mechanism, but dependent on the relative concentrations of three major reactants, i.e., RBD, mAb, and hACE2. Taken together, the IC_50_ [nM] value may depend only on the RBD or mAb spatial structure, hACE2 presence on the cell surface, which was confirmed recently [28,29], and the mAb:RBD:hACE2 ratio.

Further, comparison of the IC_50_ [nM] values from neutralization and ELISA assays with the mAb affinities to RBD determined by the SPR method using the 1:1 Langmuir interaction model demonstrated no significant differences between the IC_50_ [nM] and K_D_ [nM] values as determined by the Bland–Altman method. This finding excludes the influence of the RBD:hACE2 interaction mechanisms on the measured IC_50_ or K_D_ values. To fit binding curves with the 1:1 interaction model, we used initial concentrations of the soluble recombinant RBDs along with molecular weights obtained from the manufacturer, and we did not calculate the exact mAbs’ concentrations used for running SPR. However, since the system is reversable, one may obtain similar K_D_ values using a slightly modified RBD:mAb interaction model and ad-hoc knowledge of the bulk concentration of the tested mAbs. Since the Biacore instrument can determine specific analyte concentrations without knowledge of either analyte or ligand concentrations and since the dissociation constant K_D_ [nM] can be determined as a ratio of the rate constants K_off_ [s^−1^]/K_on_ [nM^−1^s^−1^], our data suggest that K_off_ is the single parameter that is directly associated with the mAb tertiary structure independent of reactant concentration, and appears to be the major parameter that defines the anti-RBD mAb potency. Moreover, since K_off_ can be used for calculation of the half-life of the mAb:RBD complex, this parameter can be used to predict effects of the mAb structure on the inhibition of the RBD:hACE2 interaction. It is worth noting that one of the limitations of our study is that our results were obtained using mAbs with a high affinity to the selected RBDs. We cannot exclude that if the antibodies with lower affinities (K_D_~IC_50_ >> 10 nM) are used, the cell type, pseudovirus backbone, or presence of TMPRSS2 on cell surface could start affecting the mAb potency measurements and the bias between different neutralization assays would increase significantly.

## 5. Conclusions

In conclusion, we determined the mAb potency as its concentration-independent property related to its tertiary structure conformation under the influence of physical properties of the environmental parameters it exists in. We assumed that physiological environmental parameters of the intercellular compartment are within a normal physiological range and that they do not significantly influence the spatial distribution of atomic forces on the surfaces of the interacting molecules, especially in the vicinity of the mAb paratope region, RBD epitope region, and RBD:hACE2 complex. Under these assumptions, the Bland–Altman analysis showed that IC_50_ [nM] values measured by neutralization and competition ELISA assays or K_D_ [nM] values obtained by the SPR assay are interchangeable and that the anti-RBD mAb potency can be attributed to one variable directly related to the mAb tertiary structure, i.e., the half-life of the mAb:RBD complex (T_1/2_ = ln(2)/K_off_). This variable, being dependent only on the easily experimentally obtained rate dissociation constant K_off_, can serve as a valuable new tool for potency comparison of different mAbs against a single epitope or a single mAb against different epitopes and is independent of the mechanisms of antigen–cell interactions.

## Figures and Tables

**Table 1:** Percent of pLV particles expressing a “secondary” SARS-CoV-2 variant needed to be present in the mixture to increase the IC_50_ value by 50% as compared to the “primary” SARS-CoV-2 variant.

mAbs	Mixtures of pLVs Expressing Primary:Secondary Variant ^a^		
	Wuhan:Delta	Wuhan:Omicron	Delta:Omicron
Casirivimab	N/A ^b^	≥75%:≤25%	≥96%:≤4%
Imdevimab	≥81%:≤19%	≥90%:≤10%	≥74%:≤26%
Bamlanivimab	≥88%:≤12%	≥87%:≤13%	N/A ^c^
Etesevimab	≤14%:≥86%	≥90%:≤10%	≥89%:≤11%
GenScript standard	≥46%:≤54%	≥65%:≤35%	≥21%:≤79%

^a^ “Primary” variant is the SARS-CoV-2 spike variant against which the mAb showed the lowest IC_50_ value among SARS-CoV-2 variants present in the mixture. ^b^ Neutralization titers against pLV expressing Delta variant did not differ by ≥50% as compared to those against pLV expressing Wuhan variant in 293T-hACE2 cells. ^c^ No neutralization of the SARS-CoV-2 spike Delta and Omicron variants was observed by bamlanivimab.

**Table 2:** Biases between neutralization and binding (ELISA and SPR) assays.

Binding Assays	Neutralization Assays							
	pLV				pMLV			
	293T-hACE2	293T-hACE2-TMPRSS2	A549-hACE2	A549-hACE2-TMPRSS2	293T-hACE2	293T-hACE2-TMPRSS2	A549-hACE2	A549-hACE2-TMPRSS2
**ELISA**	−0.3 ± 0.5	0.0 ± 0.9	−0.3 ± 0.5	−0.4 ± 0.5	−0.2 ± 0.6	0.0 ± 0.7	−0.3 ± 0.5	−0.3 ± 1.7
**SPR**	−0.8 ± 0.8	−0.5 ± 1.2	−0.8 ± 0.9	−0.9 ± 0.9	−0.7 ± 0.9	−0.6 ± 1.0	−0.8 ± 0.9	−0.2 ± 1.8

Note: Bias between each pair of the assays was calculated using Bland–Altman method [22] using IC_50_ [nM] values for the neutralization and ELISA assays and K_D_ values for Biacore assay. Bias between ELISA vs. SPR equals −0.5 ± 0.8 nM at 95% CI.

## Data Availability

All primary data leading to the presented figures and tables are available from the authors’ laboratory. The article reflects the views of the authors and should not be construed to represent FDA’s views or policies.
